# Atypical Imaging Features of an Incidentally Discovered Intracranial Meningioma: A Case Report

**DOI:** 10.7759/cureus.64503

**Published:** 2024-07-14

**Authors:** Nasser Alsharif, Abdulmajeed Alkhathami, Abdullah S Almalki, Shimaa s Elkholy

**Affiliations:** 1 Department of Neurosurgery, King Khaled Hospital, Najran, SAU; 2 Department of Ophthalmology, College of Medicine, University of Bisha, Bisha, SAU; 3 Department of Neurosurgery, Aseer Central Hospital, Abha, SAU; 4 Department of Pathology, Aseer Central Hospital, Abha, SAU

**Keywords:** case report, management, incidental finding, atypical imaging appearances, intracranial meningioma

## Abstract

Intracranial meningiomas are the most common primary brain tumors, typically presenting with well-defined imaging characteristics. This case report focuses on a 56-year-old female patient who was referred due to a history of head trauma and an incidental space-occupying finding to investigate the atypical imaging appearances of intracranial meningiomas, focusing on a specific case with distinct radiological findings. Meningiomas are commonly associated with specific radiological features, such as contrast enhancement, dural tail, and hyperostosis. However, this particular case exhibited atypical imaging characteristics that raised concerns about the underlying tumor type.

In-depth analysis and subsequent histopathological examination revealed a World Health Organization (WHO) grade II atypical meningioma. This variant of meningioma demonstrated increased cellularity, nuclear atypia, and a high mitotic index, indicating more aggressive tumor behavior.

The study highlights the importance of recognizing atypical imaging appearances in meningiomas, as they may indicate higher-grade tumors with a potentially different clinical course and management approach. Accurate identification of these atypical features can contribute to improved diagnostic accuracy and guide appropriate surgical decision-making for patients with intracranial meningiomas.

## Introduction

Meningioma, a common noncancerous brain tumor, can be diagnosed with greater accuracy due to the increased utilization of neuroimaging techniques. Intracranial meningiomas are generally benign tumors originating from the arachnoid cap cells surrounding the brain or spinal cord [1]. However, a subset of meningiomas may demonstrate atypical imaging appearances, which can mimic other intracranial pathologies and complicate the diagnostic process [2]. Most tumors exhibit characteristic radiological features, such as contrast enhancement, the presence of a dural tail, and hyperostosis of the neighboring bone. On computed tomography (CT), most meningiomas appear isodense to hyperdense, with approximately 20% showing calcifications.

Magnetic resonance imaging (MRI) reveals variable characteristics of meningiomas, ranging from hyperintensity to hypointensity on T1-weighted sequences and isointensity to hyperintensity on T2-weighted sequences. Meningiomas demonstrate strong enhancement on contrast-weighted images, except in areas where necrosis or cysts are present [3, 4].

This case report presents an atypical intracranial meningioma and reviews the existing literature to enhance knowledge and highlight the importance of recognizing these atypical presentations.

## Case presentation

A 56-year-old female was referred with a history of head trauma and incidental space-occupying lesion. The patient had no significant past medical history or family history of brain tumors. The neurological examination was normal. 

**Figure 1:**
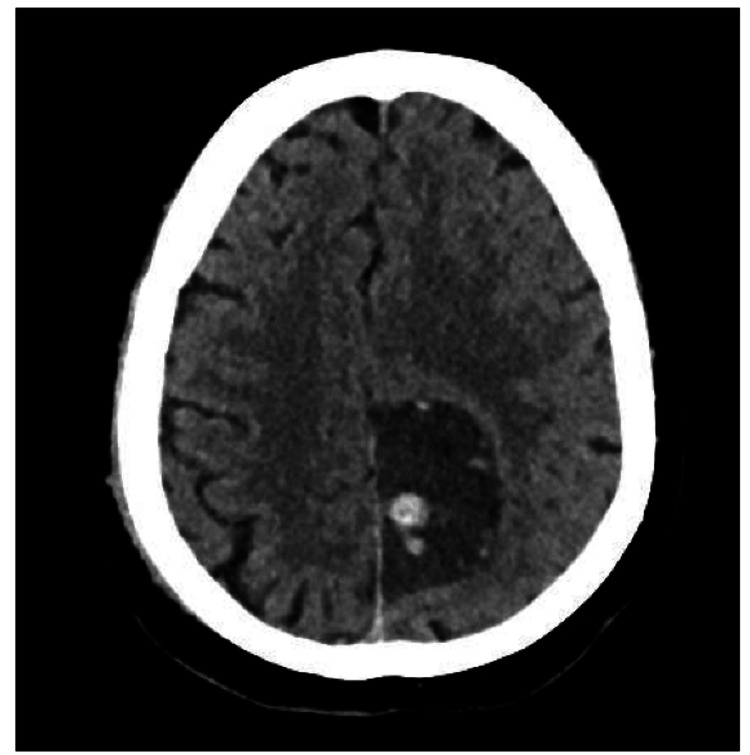
A cross-sectional CT view of the brain shows a well-defined hypodense lesion in the left parietal region, adjacent to the falx cerebri. This lesion is associated with multiple rounded, hyperdense components, most likely representing hemorrhage. There is a mass effect on the adjacent structures, although no midline shift is observed.

A CT scan without contrast was obtained and showed a left parietal parasagittal hypodense lesion with multiple hyperdense hemorrhagic components with a mass effect on the adjacent structure and no midline shift, as shown in Figure 1.

**Figure 2:**
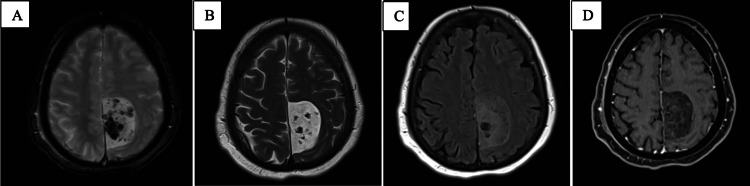
This figure comprises four MRI sequences. A: T2-weighted MRI; B: T2-weighted contrast MRI; C: fluid-attenuated inversion recovery (FLAIR) MRI; and D: T1-weighted contrast MRI. The MRI scans reveal a left frontoparietal parafalcine extra-axial lesion, measuring approximately 4.9 cm (anteroposterior, AP), 3.1 cm (transverse, TR), and 4.6 cm (craniocaudal, CC). This lesion exhibits heterogeneous signal intensities on T1 and T2/FLAIR sequences, incorporating multiple intrinsic T1 hyperintense foci that correspond to hemorrhagic densities previously observed on a CT scan. These findings are accompanied by gradient echo (GRE) bloom artifacts, heterogeneous signals on diffusion-weighted imaging (DWI), and mild to moderate heterogeneous enhancement on postcontrast sequences.

Multiplanar pre- and postcontrast-enhanced MRI sequences of the brain were obtained, revealing a left parafalcine frontoparietal extra-axial heterogeneously-enhancing mass lesion as in Figure 2. The lesion was causing a mass effect on the adjacent left frontoparietal paramedian region, the posterior body of the left lateral ventricle, and the splenium of the corpus callosum. Despite these effects, there was no significant perilesional edema or overt parenchymal enhancement, and only a mild rightward midline shift of up to 0.2 cm was noted. Additionally, a slight asymmetry in the basal cisterns was present, although there was no evidence of overt transtentorial herniation at this time. 

Surgical procedure and patient positioning

The patient underwent the surgical resection of an intracranial lesion under general anesthesia. The patient was positioned in a lateral position, and the patient's head was securely fixed in a three-pin skull clamp following induction. A left-sided fronto-parietal craniotomy was then performed to access the tumor.

Surgical approach

Using an interhemispheric approach for the tumor resection, a U-shaped skin incision was made, crossing the midline. The scalp flap was reflected inferiorly, and a craniotomy bone flap was created with a high-speed drill. During the initial exposure of the sagittal sinus, there was bleeding from the sinus, which was controlled with hemostatic agents. The dura was opened in a C-shaped fashion, allowing access to the underlying tumor. Intraoperative navigation systems were used to enhance the precision of tumor localization. 

Tumor resection

**Figure 3:**
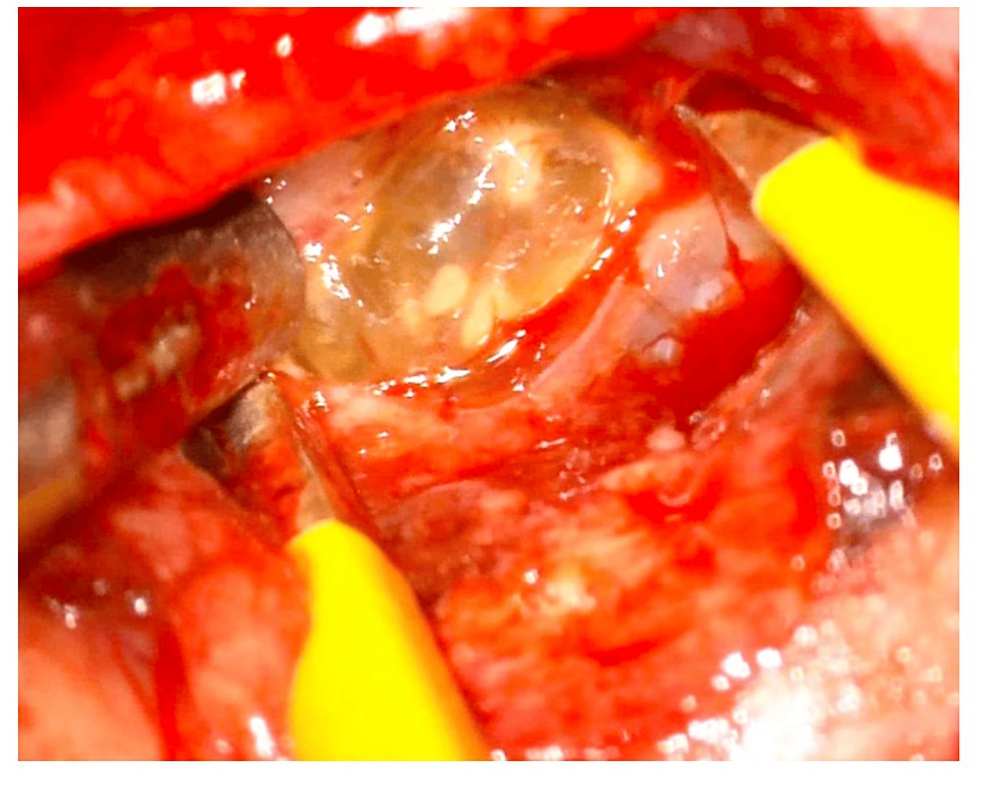
Intraoperative view of a solid and cystic brain tumor

Upon exposure, the tumor was found adherent to the falx and had invaded the adjacent brain parenchyma. Meticulous dissection was carried out to perform a totally safe resection while preserving critical neurovascular structures. The tumor was characterized by a grayish appearance, cystic components, and soft consistency, as shown in Figure 3. It was gradually debulked using suction, microsurgical tools, and ultrasonic aspirators. After tumor removal, the falx was coagulated to prevent recurrences, and the dura mater was primarily closed in a watertight fashion. The bone flap was reattached, and the surgical incision was sutured closed. The patient was then extubated and transferred to the intensive care unit for observation.

Postoperative course

**Figure 4:**
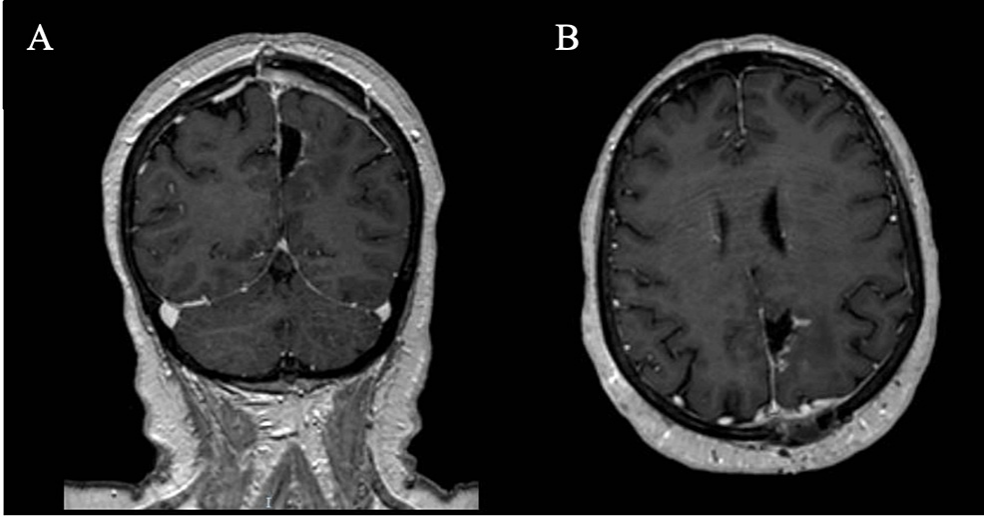
T1-contrasted MRI of the brain coronal view (A) and axial view (B) postoperatively, shows complete tumor resection.

The patient's recovery was monitored with a postoperative CT scan and MRI, as shown in Figure 4, and the patient was subsequently discharged home with continuous follow-up in the outpatient clinic.

Microscopic description

**Figure 5:**
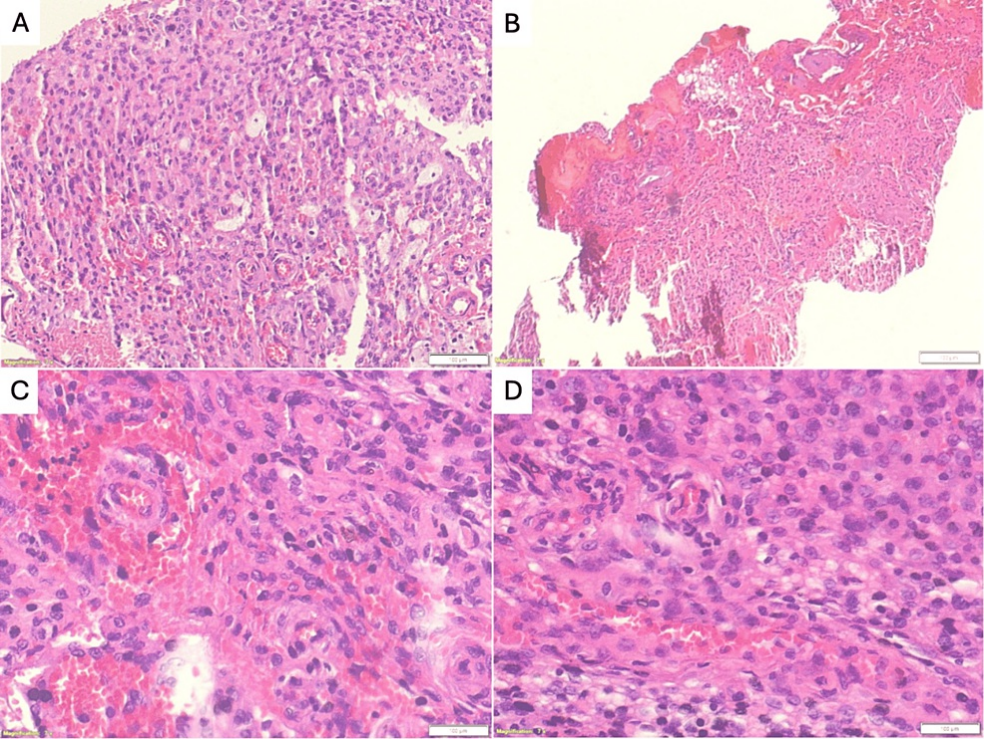
The displayed sections in (A, B, C, and D) reveal fragmented tumor tissue comprising neoplastic meningothelial cells against a collagen-rich background. Notably, the sections present focal nuclear pleomorphism and sporadic mitotic figures, signaling active cellular processes. Sparse psammoma bodies can be observed, along with regions of hemorrhage and slight necrosis within the tissue.

Sections revealed a fragmented tumor composed of neoplastic meningothelial cells with a collagenous background. Tumor cells were arranged in sheets with a focal, whorled architecture. Tumor cells exhibited eosinophilic cytoplasm and round-to-oval nuclei dispersed with chromatin. inconspicuous nucleoli and total nuclear pseudo-inclusions. Focal nuclear pleomorphism and a few scattered mitotic figures were seen. There were associated sparse psammoma bodies. Areas of hemorrhage and minimal necrosis were seen, as shown in Figure 5.

The immunohistochemistry analysis revealed that the tumor cells exhibited diffuse positive staining for epithelial membrane antigen (EMA) in approximately 80% of the tumor cell population. Additionally, the tumor cells demonstrated nuclear positivity for the progesterone receptor (PR) in 65% of the tumor cell population.

The final histopathological examination revealed a World Health Organization (WHO) grade II atypical meningioma, characterized by increased cellularity, nuclear atypia, and a high mitotic index.

## Discussion

Atypical imaging appearances of intracranial meningiomas

Atypical imaging appearances of intracranial meningiomas encompass various radiological features that deviate from the classic presentation [5]. These include cystic meningiomas, hemorrhagic meningiomas, calcified meningiomas, and meningiomas with necrosis. Each variant poses unique challenges in terms of diagnosis and management [6].

Diagnostic approaches and challenges

Diagnosing atypical meningiomas requires a multidisciplinary approach involving clinical evaluation, radiological imaging, and histopathological analysis. However, differentiating atypical meningiomas from other intracranial lesions, such as gliomas, metastases, or rare tumor types, can be challenging due to overlapping imaging characteristics. Advanced imaging techniques, such as perfusion MRI or positron emission tomography (PET), may aid in distinguishing these entities [7, 8].

Management considerations

Management of atypical intracranial meningiomas depends on various factors, including the tumor's size, location, histopathological features, and the patient's age and overall health. Surgical resection remains the mainstay of treatment, aiming for a maximally safe resection. Additionally, adjuvant therapies, such as radiation therapy or targeted therapy, may be considered in cases where complete resection is not achievable or in high-grade meningiomas [9].

Meningioma, a common tumor found within the protective layers of the brain, typically exhibits strong enhancement when contrast is used. The majority of meningiomas show either homogeneous or heterogeneous enhancement [1]. However, the particular case discussed in this report displayed a rare occurrence of faint enhancement with contrast. Additionally, classic features associated with meningiomas, such as the presence of a dural tail (found in 72% of cases) and hyperostosis (found in 50%-60% of cases), were absent in this tumor. This combination of atypical features made meningioma an unlikely diagnosis based solely on imaging findings.

Considering the tumor's typical location, minimal contrast enhancement, and hypointensity on T1-weighted images without contrast, the primary diagnosis seemed to lean towards an epidermoid cyst [4]. It is possible that a technical error during the contrast administration could explain the lack of enhancement in some cases. However, the fact that other structures, such as the nasopharyngeal mucosa, choroid plexus, and vessels, were enhanced with contrast contradicted this hypothesis. The histological examination and immunostaining confirmed that the tumor was indeed a microcystic meningioma. The tumor cells stained positive for commonly observed meningioma markers like vimentin, reticulin, and cyclin D1 [10].

An important atypical finding was the expression of glial fibrillary acidic protein (GFAP), which is primarily expressed in astrocytes rather than meningioma cells. Normally, meningioma cells do not express GFAP, except in cases involving brain invasion, where a distinct meshwork of cells is formed, consisting of meningothelial cells from the tumor interwoven with brain astrocytes expressing GFAP. In this particular case, there was no evidence of brain invasion, and GFAP expression was exclusively observed in the tumor cells. The expression of GFAP by meningothelial cells in meningioma is rare, suggesting a possible distinct precursor cell type associated with this microcystic meningioma variant, different from the precursor cells typically seen in classic meningiomas [11].

There are 15 histo- and cytomorphological varieties of meningiomas; nine of these variants correlate to WHO grade I, three to WHO grade II, and three more to the malignant form of WHO grade III meningiomas [12]. The age-adjusted incidence rate of WHO grade II meningioma is 0.26/100,000 for men and 0.30/100,000 for women [13]. Atypical meningiomas display a denser aggregation of cells compared to typical meningiomas. This increased cellularity is often evident in tightly packed cells with less intercellular space. These tumors exhibit notable nuclear variability, which includes irregular nuclear contours, variation in nuclear size (pleomorphism), and prominent nucleoli. Nuclear atypia is a marker of abnormal cell growth and division, reflecting underlying genetic changes. A significant feature of grade II meningiomas is a higher number of mitotic figures per high-power microscopic field. A mitotic count of four or more per 10 high-power fields (HPFs) is typically used as a threshold for atypical meningiomas, indicating a higher proliferative activity [14, 15]. This case emphasizes the importance of considering microcystic meningioma as part of the differential diagnosis for non-enhancing cerebellopontine masses with intermediate or no signal on diffusion-weighted imaging. Regardless of the specific tumor type, the primary goal of treatment remains a maximally safe surgical resection.

## Conclusions

The case presented demonstrates how atypical imaging features of intracranial meningiomas can create diagnostic challenges, as these tumors may resemble other types of intracranial pathologies. Accurately diagnosing and appropriately managing meningiomas with unusual radiographic appearances requires a collaborative effort between various healthcare professionals, including clinicians, radiologists, and pathologists. This case underscores the critical need for clinicians to maintain a high index of suspicion for atypical meningioma presentations and to customize treatment strategies accordingly. Recognizing the diverse spectrum of meningioma imaging characteristics is essential for ensuring timely and accurate diagnosis, which in turn enables the implementation of the most suitable therapeutic approach for each patient.
